# Enhancing GUI test case generation with multi-objective quasi-oppositional genetic sparrow

**DOI:** 10.1038/s41598-025-31221-9

**Published:** 2025-12-05

**Authors:** N. Jayalakshmi, K. Sakthivel

**Affiliations:** 1 Department of Computer Applications, PSNA College of Engineering and Technology, Dindigul, 624622 India; 2 DEPARTMENT OF COMPUTER SCIENCE & BUSINESS SYSTEMS, K.S. RANGASAMY COLLEGE OF TECHNOLOGY, TIRUCHENGODE, 637215 India

**Keywords:** Graphical user interfaces (GUI), Quasi-oppositional genetic sparrow search algorithm (OOGSSA), User friendly, Search-based software testing (SBST), Sparrow search algorithm (SSA), Computational biology and bioinformatics, Engineering, Mathematics and computing

## Abstract

To achieve robust and user friendly software, it is crucial to make sure that Graphical User Interfaces (GUI) is of quality and reliable. The paper suggests a new method of Quasi-Oppositional Genetic Sparrow Search Algorithm (OOGSSA) of generating test cases efficiently in GUI. The ultimate goal is to have multi-objective maximization through various goals of maximum test coverage, reduced redundancy and enhanced fault detection. It is an upgrading of the Sparrow Search Algorithm to combine the imitation of elite opposition based learning with genetic evolution in order to improve the population diversity and convergence rate. The suggested method automatically examines the interactions among GUI events and refines the obtained test suite with the help of adaptive learning. Oogssa is experimental evaluated, with a test suite size of 75, mouse event coverage of 95 and through various test cases with Jaccard Similarity Index (0.75–0.82) and DiceJaroWinkler Dissimilarity (0.18–0.31). OOGSSA has better adaptability which is intelligent as compared to traditional tools of automation testing, dynamic test generation and Non-manual coverage. Notice however its possibility to be costly in terms of computation in the event of very complicated GUI structure. Altogether, OOGSSA offers a scalable and intelligent scheme to test GUI and improve reliability and efficiency in today software.

## Introduction

Graphical User Interface have transformed the interaction of users with the computers and electronics. The GUI introduced visual representation of a system through visual display of information and functions through windows, icons, buttons and menus to enable immense accessibility and usability in different people with different technical backgrounds. Compared to the traditional text-based interfaces where one has to memorize commands or go through a complicated menu, GUI provide a more intuitive and interactive interface where users can follow commands with a few clicks or taps. In desktop operating systems such as windows and macOS as well as other web browsers and software applications, it is best to mention that GUI have found their way into modern computing aiding the users to navigate and interact with as many software and hardware devices with ease^1^.

GUI removes the conventional boundaries, provides easy to access multipurpose multimedia environments that facilitate better user interactions of any operating system, software apps, websites, and embedded systems. Although they are generally adopted, the design of effective GUI demands a keen attention to aspects in design like color scheme, layout, typography, and user feedback to relevance to a smooth and interactive user experience. All in all, GUI have played a pivotal role in democratizing their access to technology and giving the users the ability to make their computing environments more personalized to fit their preferences and work processes. The appearance of icons, themes, wallpapers and even the arrangement of menus and tool bars can often be customized by the users.

Normally GUI consists of several layers of software architecture which consist of presentation layer, application layer and operating system services. The presentation layer is made up of graphical objects like windows, icons, menus and widgets and displayed with help of graphical libraries and frameworks^2^. These components are manipulated and arranged by the application layer where the user input is interpreted, the commands processed and the graphical interface updated. The services of operating systems offer low level services which include window management services, event processing services and device input/output services. The interface of any type of GUI takes into consideration the concepts of human-computer interaction, psychology and design in order to maximize usability, accessibility and user experience.

**Fig. 1:**
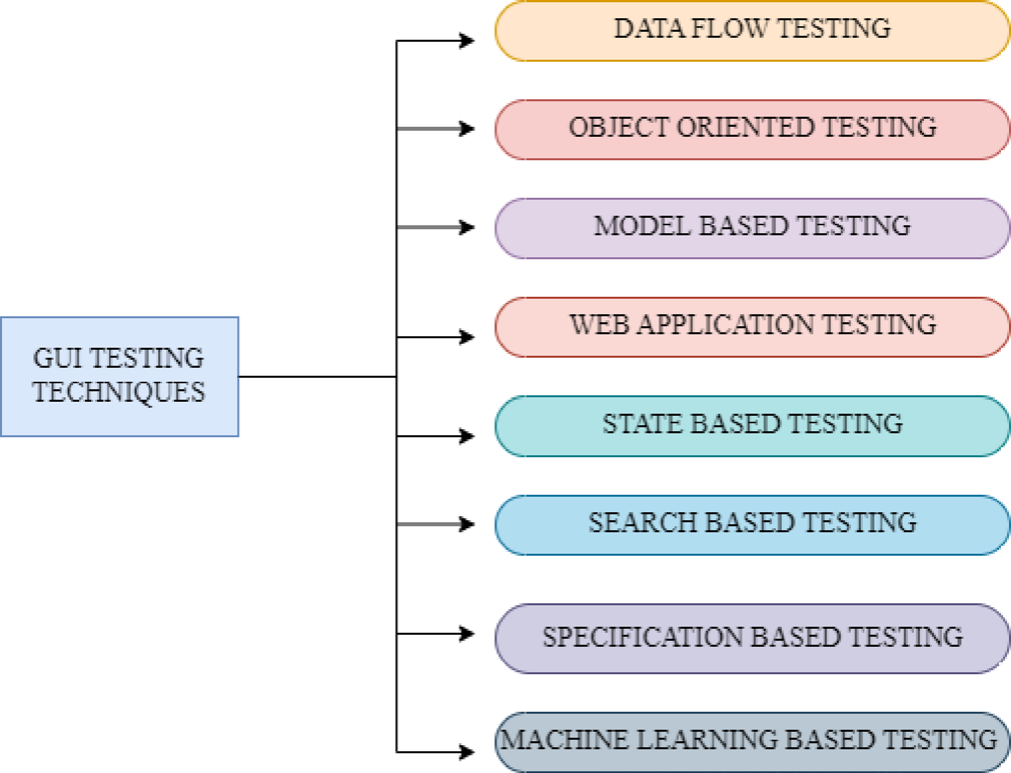
Testing Techniques.

The four components of a complete GUI environment include a User Interface (UI) style guide, graphics library, a UI toolkit, and consistent applications.An advanced programming interface for graphics is offered by the graphics library. Application programs can create and manage the conversation elements of the Icons, Windows, Pointers, Menus, and Scroll Bars (WIMPS) interface with the help of the UI toolkit, which is created within the graphics library. Applications can use the dialogue elements in a way that gives the user a uniform, intuitive experience a “look and feel” by following the guidelines stated in the UI style guide. In recent years, GUI testing has emerged as a critical element in the processing of app development, and associated research has become increasingly active^3^ GUI testing approached from several angles, such as test coverage, test case creation, test oracle, and regression testing; it is not an isolated task. It validates test events and data as well. Every GUI program has interactive elements. Verifying the parts and their interactions is therefore crucial. The triggering events are mouse clicks, graphical object selection, menu item selection, and window closure. Figure 1 illustrates different techniques of testing.

UI testing, which is an important part of software testing and emphasizes on testing the functionality, usability, and reliability of a GUI application? In this testing process, the testing team will check the interface components of the application including the buttons, menu, form, and the dialog box to make sure that they perform as per the expectations and meet the design specifications. Under GUI testing there are various tests such as functional testing where is the management of every GUI element to verify that it has your desired functionality that is correct and compatibility testing where the management of the application is to check that it functions correctly with various operating systems, browsers, and resolutions. The other tests are the usability testing which is used to test the functionality and ease of use of the application. The role of GUI testing is crucial in the detection of errors, usability problems and inconsistencies during the user interface and eventually leads to improvement of the quality and success of the entire software application^4^.

The valuable input of the proposed project is:

- A novel model for test case generation for GUI.
- A novel model that effectively generate test cases using a multi objective concept.
- A hybrid model for enhancing the GUI testing process.

The remaining portions of the paper are arranged as follows: In Sect. 2, a summary of literature is provided, highlighting areas that indicate a need for more investigation. The methodology has been described in detail in Sect. 3. The 4th section is quite detailed regarding the results that the proposed strategy had. Lastly, a conclusion is provided to the paper, and this is in Section five in which it summarises the findings.

## Literature review

The interactive aspect of the modern software has made the Graphical User Interface (GUI) testing a sensitive area as far as software quality assurance is concerned. Initial research has placed emphasis on the structure and functionality of GUIs and the issues of manual validation^1^. Principles of cognitive science have also been used in design and testing of GUI to enhance the usability and error detection^2^. Recent works have also investigated the use of combinatorial optimization methods to enhance the design and evaluation speed of GUI^3^.

Mobile and web applications have become widespread and automated GUI testing is now becoming a must. Automated Android tools have been evaluated as part of benchmarking against bugs in the real world^4^ and intent-based methods have been used to generate test cases though the use of autonomous agents^5^. The compromise between maintainability and coverage revealed by scripted and scriptless testing techniques of web applications, and the extensive presentations by systematic mapping studies of mobile GUI testing techniques^8^. A research has also been done on the industrial application of search-based GUI testing with successful results in showing its relevance in practice^9^.

Test generation is optimized through the use of search-based software engineering (SBSE) techniques. Test efficiency, fault detection, and event sequence reduction are all enhanced by metaheuristic techniques like genetic algorithms, hyper-heuristics, and swarm intelligence^10–12^. The vulnerability of scripted GUI tests is demonstrated by empirical research on Android applications, which also emphasizes the necessity of flexible and reliable test generation techniques^13^. Structural test data generation and automated test suite creation have made use of metaheuristic algorithms such as artificial bee colony, firefly, and imperialist competitive optimization^24–26^.

In addition to GUI testing, optimization and learning-based techniques have been used to improve network performance, resource allocation, and system reliability. Adaptive storage allocation in mobile networks^17^ and intrusion detection^16^ have shown the efficacy of ensemble learning and reinforcement learning techniques. For mobile ad hoc networks, QoS-aware routing and clustering techniques increase efficiency and dependability in dynamic situations^18,20^. Automated web service discovery is improved by semantic matching methods^19^. It has been demonstrated that integrating residual error modeling, fault removal efficiency, and human dynamics into software reliability greatly enhances system performance and reliability growth prediction^21–23^. The benefits and drawbacks of the current test generators are compiled in Table 1.

**Table 1:** Advantages and Disadvantages of Existing Works.

Ref	Method	Main Advantages	Main Disadvantages
^4,5^	Automated/Intent-driven GUI testing	Reduces manual effort; can generate realistic test cases; suitable for mobile apps	May produce redundant tests; dependent on tool capabilities; may not cover rare event sequences
^6,7^	Scripted and Scriptless GUI testing	Scriptless testing reduces maintenance; scripted testing offers precise control	Scripted tests are fragile and require maintenance; scriptless may miss edge cases
^8^	Systematic mapping of GUI testing techniques	Comprehensive coverage of techniques; identifies gaps in research	Does not propose new test generation method; more descriptive than practical
^9^	Industrial SBSE-based GUI testing	Can be applied in real-world settings; efficient fault detection	May require high computational resources; tuning of parameters needed
^10–12^	Search-based software testing (GA, hyper-heuristics)	Improves coverage and fault detection; flexible to multiple objectives	Can be computationally expensive; results depend on fitness function design
^13^	Scripted Android GUI testing	Provides historical evolution insights; helps understand fragility causes	Fragile to app updates; maintenance-heavy
^24–26^	Metaheuristic/swarm intelligence (ABC, Firefly, ICO, SSA)	Efficient in multi-objective optimization; adaptable to various testing tasks	Requires careful parameter tuning; may converge to local optima
^16–20^	Learning-based/Network-inspired optimization	Improves reliability, resource allocation, and adaptability	Not directly focused on GUI testing; complex models may be hard to integrate
^21–23^	Software reliability growth modeling	Accounts for human dynamics and fault removal efficiency; enhances prediction accuracy	May be theoretical; limited direct impact on GUI test generation

The development of a multi-objective quasi-oppositional genetic sparrow algorithm to improve GUI test case generation while balancing coverage, fault detection, and execution efficiency was prompted by these studies’ collective emphasis on the benefits of combining multi-objective optimization, metaheuristic search, and dynamic modeling principles.

The need for more effective and efficient methods in GUI testing to handle the difficulties related to the complex structure of GUI is one research gap that is revealed by the current literature. Even though current research has made great progress in automating GUI testing procedures, there is still a noticeable gap in fully addressing the complexities of GUI. In particular, methodologies or frameworks that can more precisely and thoroughly verify the correctness of GUI are still needed, even with the progress made in automated GUI testing tools and techniques. Due to the delicate nature of GUI interactions, manual testing is still frequently relied upon despite being error-prone and time-consuming. Existing automated testing approaches often struggle with capturing all possible test scenarios and ensuring thorough coverage of GUI functionalities. Therefore, there is a need in the development of techniques that can effectively address the complexities of GUI, ensuring comprehensive testing while minimizing manual intervention and the likelihood of overlooking critical test scenarios.

## Materials and methods

**Fig. 2:**
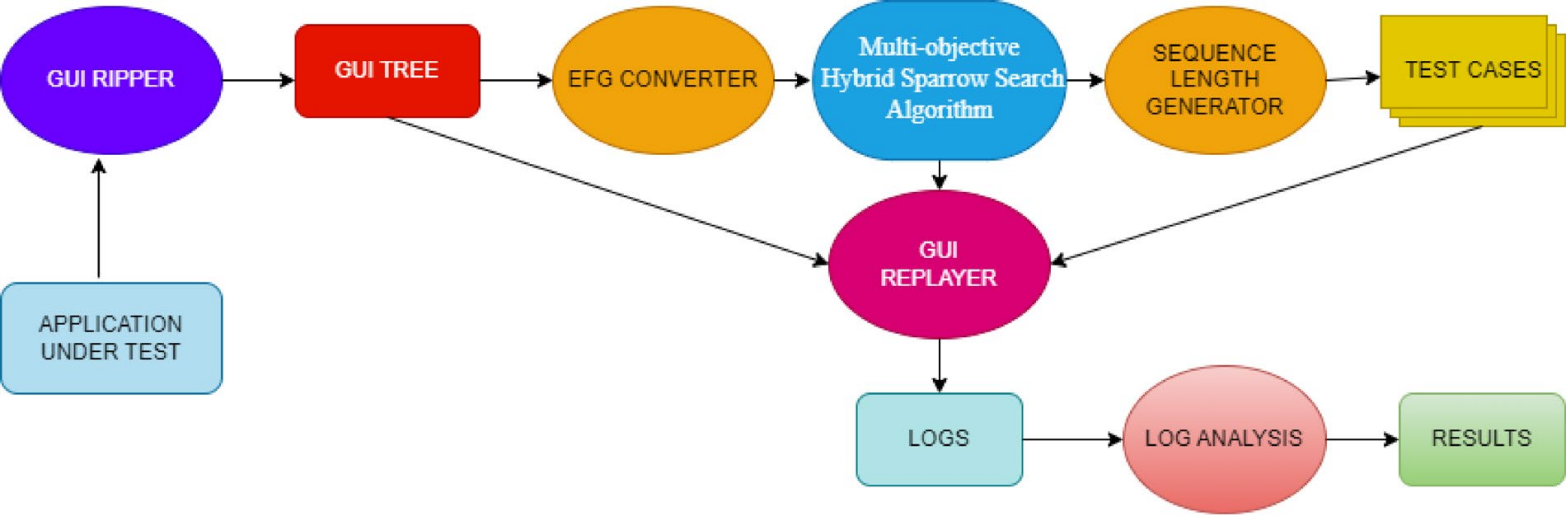
Standard workflow using PyAutoGUI.

Test case generation for GUI applications adheres to functional specifications to cover multi-way interactions between GUI model events. In addition to meet functional requirements, the generated test cases must also consider non-functional objectives such as cost-effectiveness to enhance the robustness of the testing tool. Hence, a search-based software testing (SBST) approach is proposed to produce dependable test cases for GUI applications. The standard workflow of the proposed model is illustrated below in Fig. 2.

### GUI ripper

The application under test (AUT) for GUI testing which refers to the software application undergoing evaluation to ensure its GUI functions as intended is given to GUI ripper. It extracts information about its GUI. This information typically includes the structure of GUI elements such as windows, buttons, menus, and their relationships. All the widgets and properties of widgets are also extracted from the GUI which include attributes like size, colour, position. The information about the widgets like whether it closes a window and whether it is a button or text form are also extracted. The GUI Ripper initiates the application, detects its main windows and initial GUI configurations, triggers all visible events, and proceeds to recognize any additional windows that may emerge. This process of event execution and GUI state extraction persists until the GUI has been exhaustively explored. After the structural data has been extracted, it is stored in a data structure called a GUI tree.

#### Event flow graph converter

**Fig. 3:**
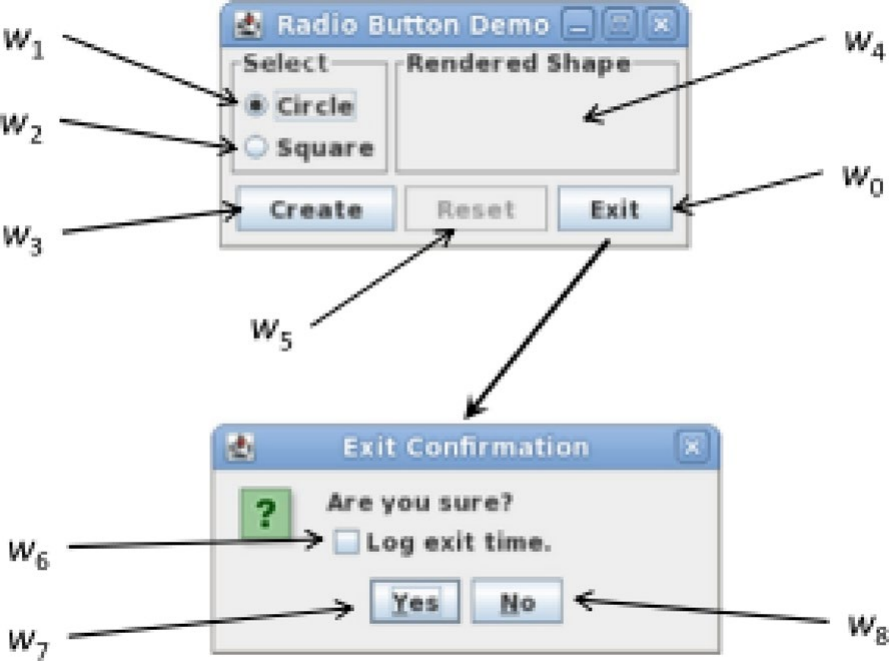
GUI application example.

The GUI Ripper outputs a GUI tree representing the structure of the application’s user interface. This tree captures the hierarchy of GUI components and their properties, forming a comprehensive model of the application’s GUI. The GUI Tree model, produced by the Ripper, can be transformed into a graph showing the relationships between events in the application’s graphical user interface (GUI) using the platform-independent framework offered by the Graph Converter. The framework facilitates the processing of the GUI Tree input and produces a graph that is used to create test cases. Figure 3 illustrates a simple GUI application with only two windows, one is a top-level window and other is a modal window, that is with exit confirmation.

**Fig. 4:**
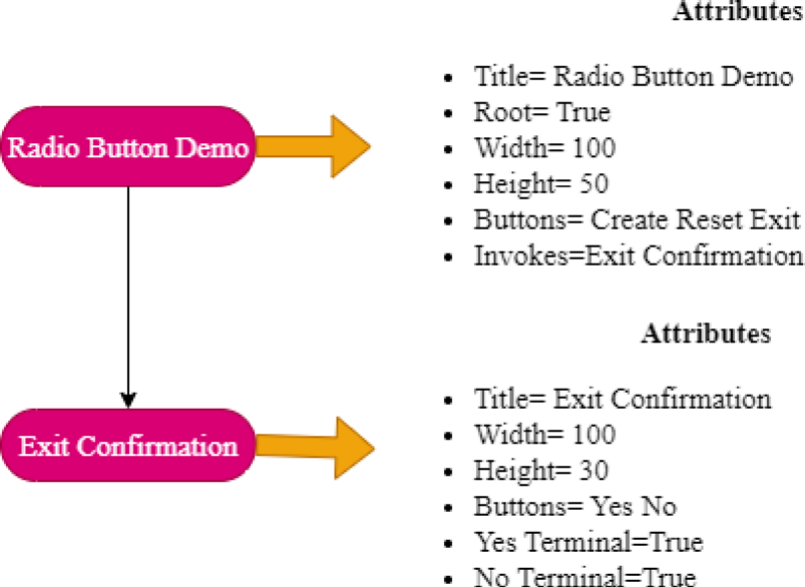
GUI Tree.

The widgets used for the application is given from $\:{w}_{0}$ to $\:{w}_{8}$. With the exception for $\:{w}_{4}$ which only display GUI information, all other widgets allow for the execution of GUI event. Figure 4 shows the GUI tree of the GUI application shown in Fig. 3 having two nodes with subset of their attributes.

**Fig. 5:**
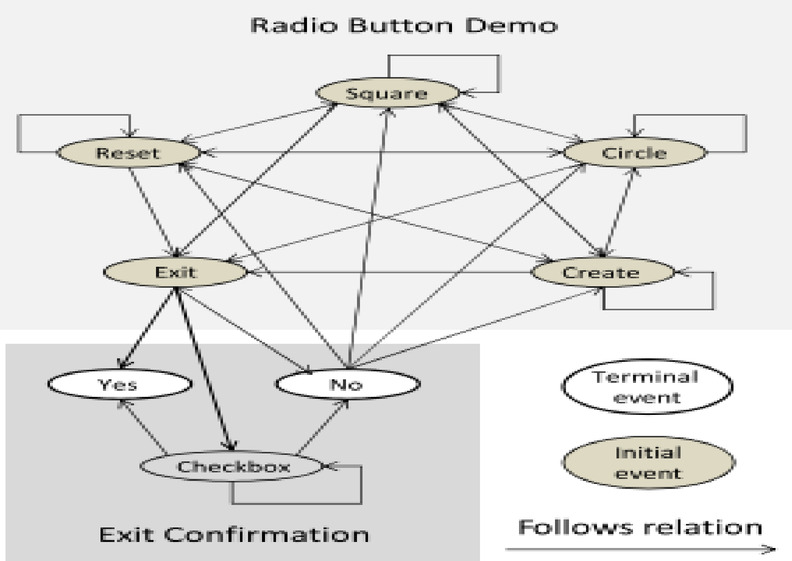
Event Flow Graph.

The events and the sequences which can executed on a GUI of a GUI based application is represented with the help of a GUI model termed Event Flow Graph (EFG). An event executed on the GUI is represented by a vertex in the directed EFG. When an edge $\:{e}_{i}\to\:{e}_{j}$ connects vertex *i* to *j*, that is that event *j* executes right away following event *i.* There is a claim that event *i* comes first. The EFG intuitively simulates several GUI execution paths. Technically an EFG is given by triple $\left\langle {{\text{V}},{\text{E}},{\text{B}}} \right\rangle$ where $\:B\:\in\:V$ is a set of vertices corresponding to initial events, or events that can be executed right away after the application begin; and $\:E\:\in\:V\times\:\:V$ is a set of directed edges illustrating the above relation.; *V* is a set of vertices indicating events on the GUI within a GUI-based application^14^. Figure 5 represents a EFG obtained for the GUI application on Fig. 3.

Events are represented in this image as ovals, with shaded ovals denotes the initial events and directed edges denotes the one-to-one relationship that follows. The terminal events are those that make application’s model GUI window to close. They are represented by white ovals.

### Proposed model architecture

#### Sparrow search algorithm

The Sparrow Search Algorithm (SSA) simulates the foraging behavior of sparrows, where individuals with high energy levels act as leaders (producers) guiding others (scroungers) to food sources. When a predator is detected, alarm signals prompt the producers to lead all scroungers to safety. The proportion of producers to scroungers remains constant within the population. Sparrows with higher energy become producers, while scroungers follow those providing the best food. Some scroungers may compete for food, while others move to safer areas when danger is sensed.When sparrows sense danger in the foraging area those on the outskirts swiftly head towards safety to secure a better position, while those in the center wander randomly, aiming to stay close to their flock mates^15^. In SSA consider that there are *n* sparrows in *d* dimensional search area, the position of sparrow can be given by the following matrix in Eq. (1),

1 $$\:X = \left[ {\begin{array}{*{20}c} {\begin{array}{*{20}c} {x_{{{\text{1,1}}}} } & {\:x_{{{\text{1,2}}}} } \\ {\:x_{{{\text{2,1}}}} } & {\:x_{{{\text{2,2}}}} } \\ \end{array} } & {\:\begin{array}{*{20}c} { \cdots \:} & {\:x_{{1,d}} } \\ {\: \cdots \:} & {\:x_{{2,d}} } \\ \end{array} } \\ {\:\begin{array}{*{20}c} \vdots & {\: \vdots } \\ {\:x_{{n,1}} } & {\:x_{{n,2}} } \\ \end{array} } & {\:\begin{array}{*{20}c} \vdots & {\: \vdots } \\ {\: \cdots \:} & {\:x_{{n,d}} } \\ \end{array} } \\ \end{array} } \right]$$

where $\:{x}_{n,d}$ represents the position of the *nth* sparrow in d dimension. The fitness value measurement of all sparrow is expressed by the vector matrix as Eq. (2),

2 $$\:F_{x} = \left[ {\begin{array}{*{20}c} {\begin{array}{*{20}c} {f(x_{{{\text{1,1}}}} } & {\:x_{{{\text{1,2}}}} } \\ {\:f(x_{{{\text{2,1}}}} } & {\:x_{{{\text{2,2}}}} } \\ \end{array} } & {\:\begin{array}{*{20}c} { \cdots \:} & {\:x_{{1,d}} )} \\ {\: \cdots \:} & {\:x_{{2,d}} )} \\ \end{array} } \\ {\:\begin{array}{*{20}c} \vdots & {\: \vdots } \\ {\:f(x_{{n,1}} } & {\:x_{{n,2}} } \\ \end{array} } & {\:\begin{array}{*{20}c} \vdots & {\: \vdots } \\ {\: \cdots \:} & {\:x_{{n,d}} )} \\ \end{array} } \\ \end{array} } \right]$$

The $\:{F}_{x}$ value in each of the row represents the fitness value of each individual. The higher the fitness value higher will be the probability for obtaining food during the foraging period. The position of the producer must update in each iteration since the search for food can be happen at anywhere as follows with respect to Eqs. (1) and (2) and given by Eq. (3),

3 $$\:{X}_{ij}^{t+1}=\left\{\begin{array}{c}{X}_{ij\:}^{t}.\:exp\left(\frac{-i}{\alpha\:.{iter}_{max}}\right)\:\:\:\:if\:{R}_{2}<ST\:\:\:\:\:\:\:\:\:\:\:\:\:\:\:\:\:\:(3.a)\\\:{X}_{ij}^{t}+\:Q.L\:\:\:\:\:\:\:\:\:\:\:\:\:\:\:\:\:\:\:if\:{R}_{2}\ge\:ST\:\:\:\:\:\:\:\:\:\:\:\:\:\:\:\:\:\:\:(3.b)\end{array}\right.$$

where *t* is the current iteration and $\:{X}_{ij\:}^{t}$represents the *t* iteration of ith sparrow in the *jth* dimension.$\:\alpha\:\in\:\left[\text{0,1}\right]$ is a random number $\:{Iter}_{max}$ represents the maximum iteration of the given population. $\:{R}_{2}\in\:\left[\text{0,1}\right]$ is the alarm threshold and $\:ST\in\:\left[\text{0.5,1.0}\right]$ is the safety threshold. Q is a random number following the normal distribution and L represents a $\:1\times\:d$ matrix where all the value are less than 1. The case shows that, when$\:{\:R}_{2}<ST$ then the sparrows are in safe zone and continue their search for food and when $\:{R}_{2}\ge\:ST$then a danger sign evolves by the predators and whole population should fly immediately to a safe place. Equation (3) updates the positions of producers that lead the search toward promising areas, thereby driving global exploration. Like producers, the position update of scroungers is given by Eq. (4),

4 $$\:{X}_{i,j}^{t+1}=\left\{\begin{array}{c}Q.exp\left(\frac{{X}_{Worst}^{t}-{X}_{i,j}^{t}}{{i}^{2}}\right)\:\:\:\:\:\:\:\:\:\:\:\:\:\:\:\:\:\:\:\:\:\:\:\:\:\:\:\:\:\:\:\:\:\:\:\:\:\:\:\:\:\:\:\:\:if\:i>\frac{n}{2}(4.a)\\\:{X}_{OP}^{t+1}+\left|{X}_{i,j}^{t}-\:{X}_{OP}^{t+1}\right|.{A}^{T}{\left(A{A}^{T}\right)}^{-1}.L\:\:\:\:\:\:\:\:\:\:\:\:\:\:\:otherwise(4.b)\end{array}\right.$$

where $\:{X}_{OP}$ is the optimum position of producers.$\:{X}_{Worst}$ is the current worst location of scrounger. In the case when $\:i>\frac{n}{2}$,the fitness value will be very low for the scroungers resulting in starvation. Equation (4) governs the scroungers, which exploit these regions to refine the current solutions and strengthen local search.The mathematical expression for the position of the sparrows which are aware of the predators accounts about 10% to 25% of the total population of sparrows is given by Eqs. (5),

5 $$\:{\text{X}}_{\text{i},\text{j}}^{\text{t}+1}=\left\{\begin{array}{c}{\text{X}}_{\text{b}\text{e}\text{s}\text{t}}^{\text{t}}+\beta\:\:.\:\left|{\text{X}}_{\text{i},\text{j}}^{\text{t}}-{\text{X}}_{\text{B}\text{e}\text{s}\text{t}}^{\text{t}}\right|\:\:\:\:\:\:\:\:\:\:\:\:\:\:\:\:\:\:\:\:\:\:\:\:\:\:\:\:\:\:\:\:\:\:\:\:\:\:\:\:\:if\:{\text{f}}_{\text{i}}\ne\:{\text{f}}_{\text{b}\:\:\:}(5.a)\\\:{\text{X}}_{\text{i},\text{j}}^{\text{t}}+H\:.\left(\frac{\left|{\text{X}}_{\text{i},\text{j}}^{\text{t}}-{\text{X}}_{\text{W}\text{o}\text{r}\text{s}\text{t}}^{\text{t}}\right|}{\left({\text{f}}_{\text{i}}-{\text{f}}_{\text{w}}\right)+{\updelta\:}}\right)\:\:\:\:\:\:\:\:\:\:\:\:\:\:\:\:\:\:\:\:\:\:\:\:\:\:\:\:\:\:\:\:\:\:\:\:\:\:\:\:\:\:\:if\:{\text{f}}_{\text{i}}={\text{f}}_{\text{b}}(5.b)\end{array}\right.$$

where $\:{X}_{Best}$ is current best position, $\:H\in\:\left[\text{0,1}\right]$and $\:\beta\:$ represents the step size parameter.$\:{f}_{i}$represents the fitness value of ith sparrow and $\:{f}_{b}$ and $\:{f}_{w}$ indicate the optimum best and worst fitness values accordingly.$\:{f}_{i}\ne\:{f}_{b}$represents that the sparrow is at the end of the population and $\:{f}_{i}={f}_{b}$ mention about the sparrows which are aware about the danger in the middle of the population and need to flee to a safe place. Equation (5) models the adaptive movement of aware sparrows that react to environmental danger, enhancing population diversity and avoiding premature convergence.

#### Genetic algorithm

**Fig. 6:**
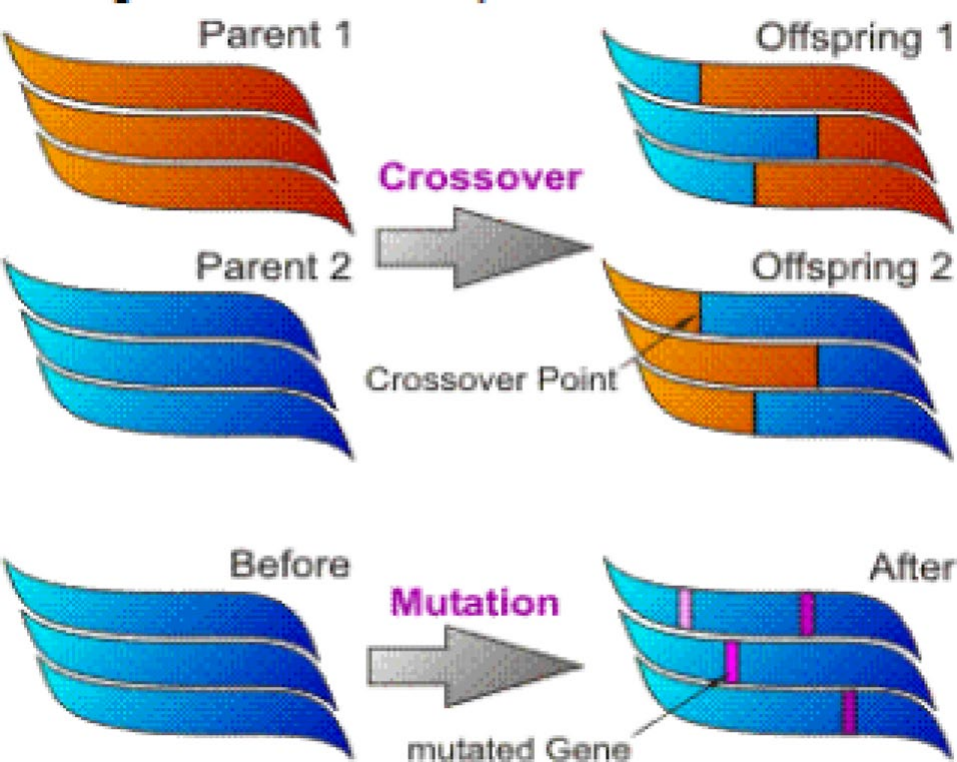
Genetic Algorithm Operator.

Darwin’s evolutionary theory served as the inspiration for the heuristic search method known as the genetic algorithm, which is an example of an evolutionary algorithm. The algorithm’s fundamental concept is to begin with a population of people that has been randomly initialized. Every person has the ability to be a viable contender for solving a particular issue. Every individual’s quality and adequacy are assessed using a fitness function. Following this, a selection procedure that is skewed toward each individual’s fitness removes a subset from the Termination criterion. Figure 6 shows the genetic algorithm operator.

Solutions are therefore more likely to be chosen. Together, these chosen people create a new population generation.Typically, a crossover operation is used to combine two people by having them exchange information at a randomly chosen place. A mutation process is frequently used to keep people from becoming too similar to one another and causing the population to freeze. The mutation operation updates a chosen individual’s information at random. Each person is reassessed following the creation of a new population, and the procedure is continued until a particular termination requirement is met.

#### Proposed quasi oppositional genetic sparrow search algorithm

The initial population’s position is initialized at random. There is no guarantee regarding the diversity or quality of the initial population produced by the random initialization procedure. As a result, the SSA solution’s accuracy drops. The initial population’s quality can be significantly raised by using the opposition-based learning method. The conventional SSA algorithm does not require any prior knowledge. In order to preserve the population’s diversity, this paper adopts an elite opposition-based learning incorporated with sparrow search algorithm for the initialization of sparrow population.

A. Elite Opposition Based Learning (EOBL).

For enhancing the diversity of the population, EOBL based on reverse learning is used. This strategy is close to the global optimum solution and hence prevent from falling into a local optimization. The algorithm is done by selecting some elite individuals for the current solution in reverse solution process in order to avoid the generation of non-elite individuals from invalid solutions. Considering $\:{x}_{i}\left(t\right)$ is the solution in *i* th iteration given by$\:{x}_{i}\left(t\right)=\left[{x}_{i1},{x}_{i2},....{x}_{id}\right]$ and the opposite solution is represented by $\:{x}_{i}^{-}\left(t\right)$.The elite individuals by considering the size $\:\rho\:(0<\rho\:\le\:n,\rho\:\in\:N\:\:$are defined by Eq. (6) as follows

6 $$\:{N}_{p}\left(t\right)=\left[{N}_{1}\left(t\right),{N}_{2}\left(t\right),.....,{N}_{p}\left(t\right)\right]$$

where $\:{N}_{p}\left(t\right)$ and n gives total solution. In d dimension, $\:p\:={x}_{a},{x}_{b}.....{x}_{d}$,then the opposite point represented by $\:{p}^{\star\:}=\left[{x}_{a}^{\star\:}{x}_{b}^{\star\:}{....x}_{d}^{\star\:}\right]$ is find out by Eqs. (7),

7 $$\:{x}_{k}^{\star\:}={k}^{{\prime\:}}\times\:({a}_{k}+{b}_{k})-{x}_{k}$$

where $\:{k}^{{\prime\:}}\in\:\left[\text{0,1}\right]$. The minimum optimization problem based on opposition is given by fitness function (*f).* If $\:f\left(X\right)\:<f\left({X}^{{\prime\:}}\right)$,the reverse solution $\:{X}^{{\prime\:}}$ is replaced by the viable solution $\:X$. The EOBL solution is given by Eq. (8) as,

8 $$\:{x}_{i,k}^{*}\left(t\right)={k}^{{\prime\:}{\prime\:}}\times\:\left({a}_{k}\right(t)+{b}_{k}(t\left)\right)-{x}_{i,k}\left(t\right)$$

where $\:{k}^{{\prime\:}{\prime\:}}\in\:\left[\text{0,1}\right]$,$\:{a}_{j}\left(t\right)$ and $\:{b}_{j}\left(t\right)$ represents the min and max of $\:{N}_{1j}\left(t\right),{N}_{2j}\left(t\right)....{N}_{\rho\:j}\left(t\right)$ The algorithm for opposition learning strategy with SSA is given below.

**Algorithm 1:**
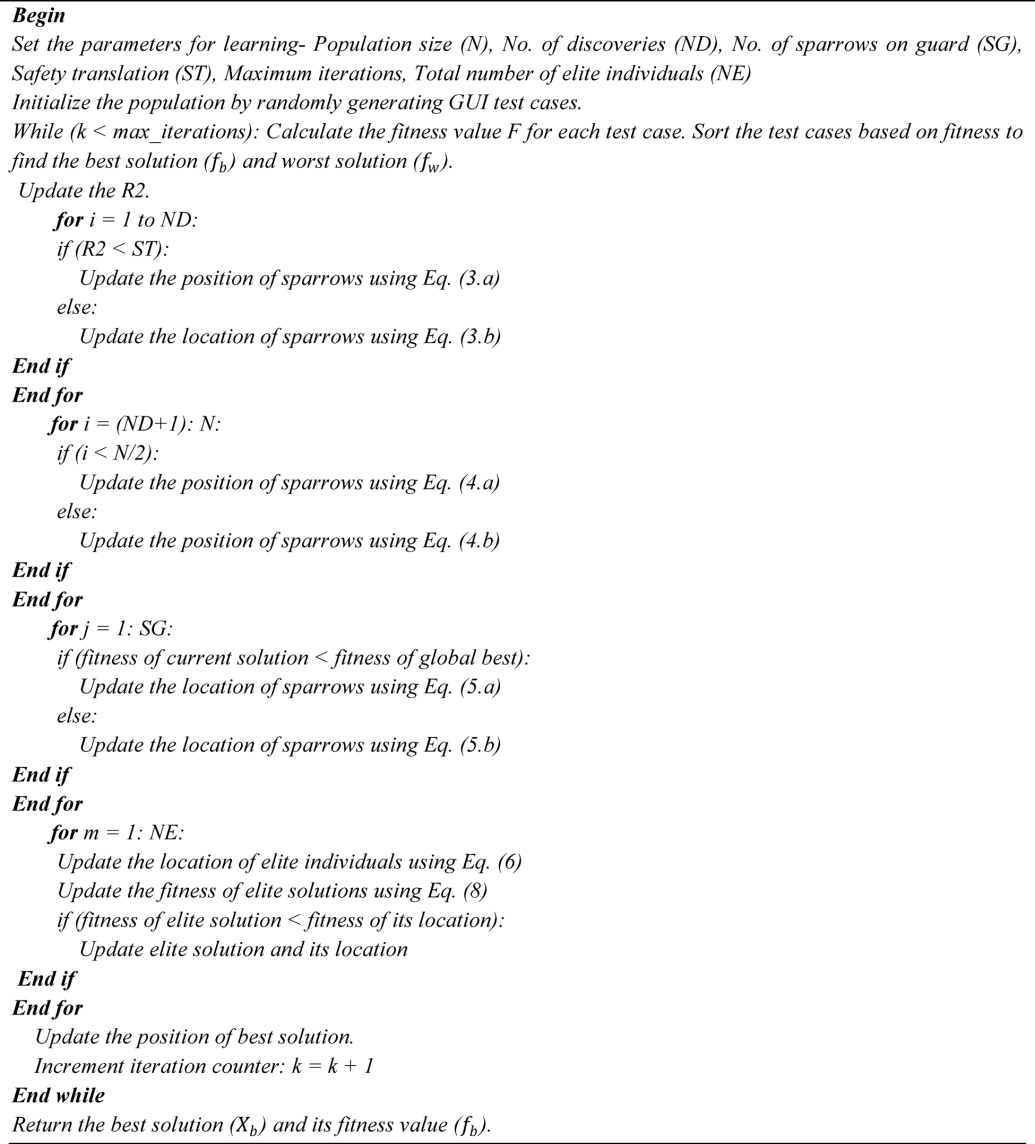
Pseudo Code of Opposition based learning with SSA.

B. GA in SSA.

**Fig. 7:**
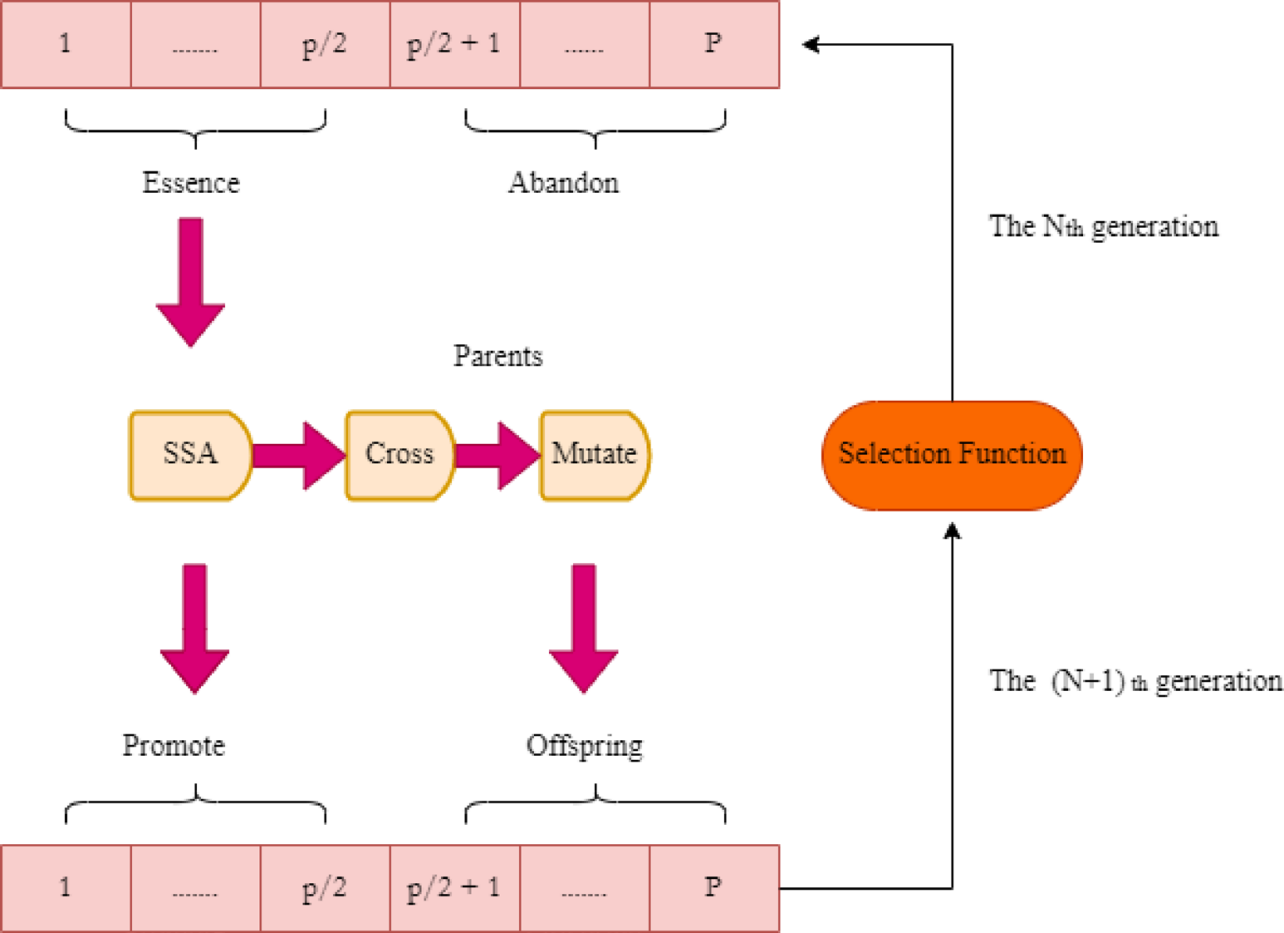
Genetic Algorithm in Sparrow Search Algorithm.

The genetic algorithm introduced in sparrow search algorithm is illustrated in the flowchart in Fig. 7. In SSA, by sharing and self-learning information between the sparrow individuals enhance the quality of population. Through cross over mutation better future population can be generated. The global search property of GA incorporates with the position transfer method in SSA. The information that gets ignored by the population in GA is fully utilized for the optimization efficiency. In addition, one of the key elements influencing the genetic algorithm’s capacity for optimization is the choice of crossover probability $\:({p}_{c}$) and mutation probability $\:\left({p}_{m}\right).$A $\:({p}_{c}$) that is too small will cause the generation rate of new individuals during iteration to slow down, which will cause the calculating process to end early. An excessively large $\:({p}_{c}$) can lead to an excessive number of recently generated individuals in the group, thereby undermining the quality of previously generated great individuals. The potential to create new individuals via mutation functions will be limited if $\:\left({p}_{m}\right)$ is too short, and many good genes being lost too soon and not make it into the next generation, therefore being counterproductive to preserving population variety. Lastly, it resembles a random search process if $\:\left({p}_{m}\right)$ is excessively large. Also proposes a new cross over and mutation update based on golden ratio index by determining the proportions of crossover and mutation rates for making the individuals through particular evolution also changes.

In GA-SSA, the chromosomes in GA are depicted as sparrow in SSA. In GA, a single sparrow in SSA is viewed as a chromosome. After being enhanced, crossing across, and mutating, individual sparrows in the Nth generation group go into the (*N* + 1)-th generation. For introducing GA in SSA, enhance the selection operator by calculating the fitness value of each individual sparrow in each generation, sorting the results based on fitness value, and selecting the top half of the best individuals as great samples to be further refined using the SSA algorithm to move on to the next generation.Crossover mutation function choose exceptional members of the SSA population to be parents in order to get remaining *p/2* next-generation individuals. Then, using dynamic crossover and mutation operators, create new offspring individuals for the next generation. The improvised equation of probability of cross over and mutation is expressed mathematically as Eqs. (9) and (10),

9 $$\:{p}_{c}=\left\{\begin{array}{c}{p}_{cm}\left\{exp(-0.618)\frac{{f}^{{\prime\:}}-{f}_{average}}{{f}_{max}-{f}_{average}}\right\},\:\:\:\:\:\:\:\:\:\:\:\:\:\:\:\:\:\:\:\:\:\:\:\:\:\:\:{f}^{{\prime\:}}\ge\:{f}_{average}\\\:{p}_{cm},\:\:\:\:\:\:\:\:\:\:\:\:\:\:\:\:\:\:\:\:\:\:\:\:\:\:\:\:\:\:\:\:\:\:\:\:\:\:\:\:\:\:\:\:\:\:\:\:\:\:\:\:\:\:\:\:\:\:\:\:\:\:\:\:\:\:\:\:\:\:\:\:\:\:\:\:{f}^{{\prime\:}}\le\:{f}_{average}\end{array}\right.$$

10 $$\:{p}_{m}=\left\{\begin{array}{c}{p}_{mm}\left\{exp(-0.382)\frac{{f}_{max}-{f}^{{\prime\:}}}{{f}_{max}-{f}_{average}}\right\},\:\:\:\:\:\:\:\:\:\:\:\:\:\:\:\:\:\:\:\:{f}^{{\prime\:}}\ge\:{f}_{average}\\\:{p}_{mm},\:\:\:\:\:\:\:\:\:\:\:\:\:\:\:\:\:\:\:\:\:\:\:\:\:\:\:\:\:\:\:\:\:\:\:\:\:\:\:\:\:\:\:\:\:\:\:\:\:\:\:\:\:\:\:\:\:\:\:\:\:\:\:\:\:\:\:\:\:\:\:\:\:\:\:{f}^{{\prime\:}}\le\:{f}_{average}\end{array}\right.$$

where $\:{p}_{cm}$is the max crossover probability and $\:{\:\:p}_{mm}$ is the max mutation probability and their values are set to 0.7 and 0.01 respectively. Equations (9) and (10) introduce adaptive crossover and mutation probabilities derived from the Genetic Algorithm component. These operators enable information exchange and controlled randomness, respectively, ensuring that the algorithm maintains a balance between intensification and diversification.The introduction of golden ratio also enhances the global optimum solution speed. The algorithm for the improved SSA with GA is below:

**Algorithm 2:**
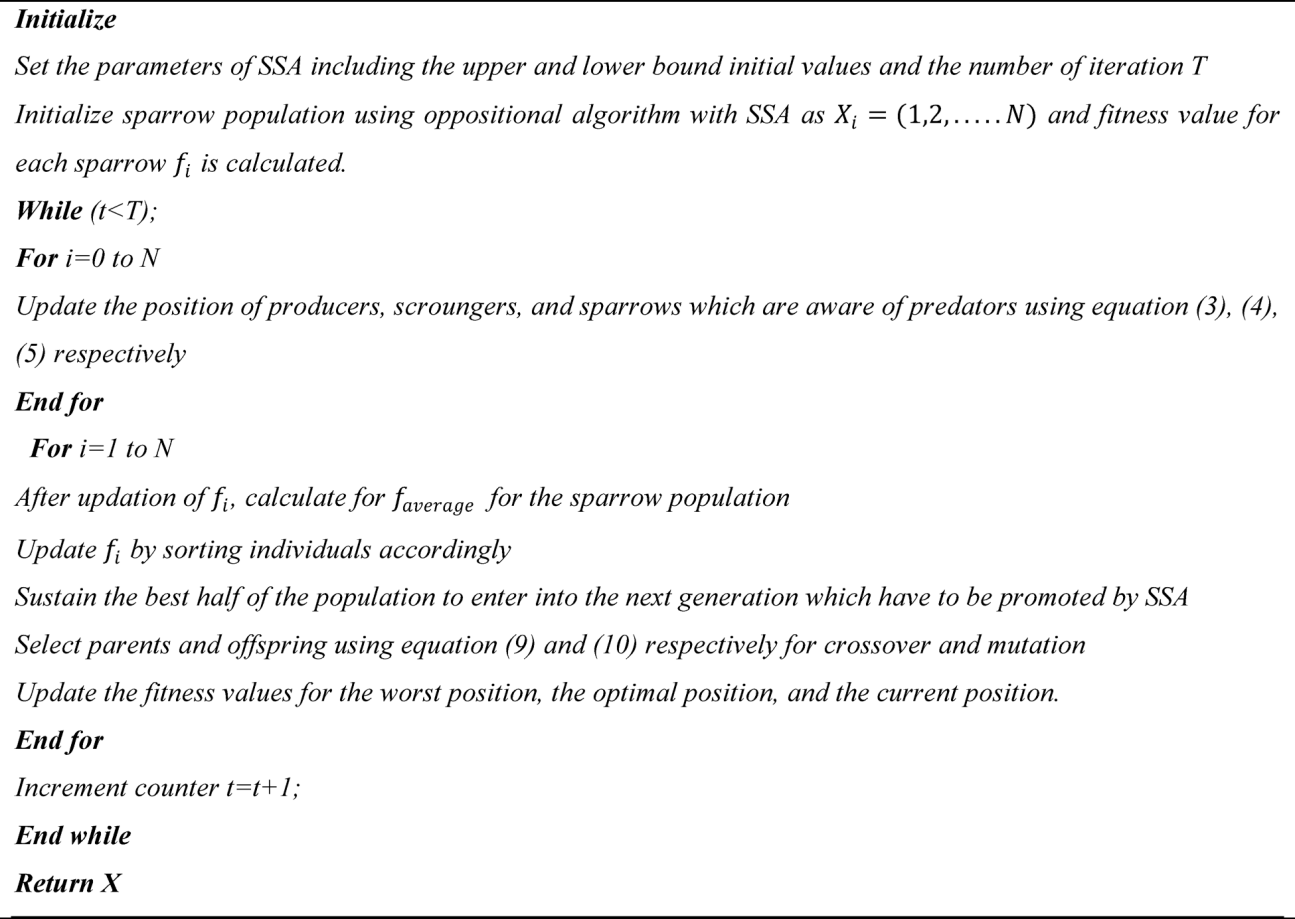
Pseudo Code of GA with SSA.

After the execution of the quasi-opposition genetic sparrow search algorithm, the sequence length is generated for the solution for the effective test case generation and given to the log analysis for the evaluation.

### Overall workflow of the proposed OOGSSA-Based GUI test case generation method

***Start***

Input Application Under Test (AUT)

- GUI model extraction begins.

GUI Ripper

- Extracts GUI components (windows, buttons, menus, etc.).
- Builds GUI Tree structure

Event Flow Graph (EFG) Converter

- Converts GUI Tree → Event Flow Graph (EFG).
- Represents all possible event sequences

Population Initialization

- Generate initial test cases (event sequences).
- Apply Elite Opposition-Based Learning (EOBL) to enhance population diversity.

Fitness Evaluation

- Assess test cases by multi-objective metrics:
  - Test coverage.
  - Diversity.
  - Test suite size.

Optimization Using OOGSSA.

- Sparrow Search Algorithm (SSA): Global search.
- Genetic Operators (GA):
  - Crossover and mutation (based on golden ratio adaptation).
- Opposition Learning: Avoids local optima.

Update Population.

- Replace weak individuals with elite or opposite solutions.

Convergence Check.

- If fitness criterion not met → continue iterations.

Generate Optimized Test Suite.

- Final diverse test cases with maximum GUI coverage.

Log Analysis & Result Evaluation.

- Compute metrics: Test suite size, Jaccard Similarity, Dice–Jaro–Winkler Dissimilarity, Event Coverage.

***End***

Programming language independence allows the suggested Quasi Opposition-based Genetic Sparrow Search Algorithm (OOGSSA) for GUI test case generation to be used in a variety of software environments. OOGSSA works by interacting with the graphical user interface at the event level—through actions like mouse clicks, keystrokes, and button activations—instead of relying on internal program logic or syntax, in contrast to conventional testing methods that demand access to source code. As universal abstractions shared by various interface frameworks, test cases are created from GUI components and the event sequences that correspond to them. The approach can be used with applications created in heterogeneous ecosystems like Java Swing, NET Windows Forms, or web technologies utilizing HTML and JavaScript because it depends on GUI models or event-flow graphs that represent user interaction patterns. The ability to programmatically access or model the GUI is the fundamental prerequisite, allowing the algorithm to be flexible across various platforms and implementation languages. As a result, OOGSSA’s language-agnostic design guarantees its wide applicability for automated testing in multi-technology software projects, improving cross-platform integration, scalability, and maintainability in contemporary software quality assurance procedures.

Table 2 lists the essential calibration parameters for the quasi Opposition-based genetic sparrow search algorithm (OOGSSA) for effective GUI test case generation

**Table 2:** Calibration parameters.

Parameter	Symbol	Typical Value	Description
Population Size	N	30	Number of candidate solutions (sparrows) in the population per iteration.
Number of Search Agents	–	50	Total number of sparrow individuals exploring the search space.
Safety Threshold	ST	0.6	Threshold for distinguishing safe and danger zones in sparrow behavior modeling.
Alarm Threshold	R2	Dynamic	Random alarm trigger level determining escape behavior in sparrows.
Number of Discoverers	ND	0.4 × N	Proportion of sparrows designated as producers searching for food (best solutions).
Number of Guards	SG	0.3 × N	Proportion of sparrows guarding against danger (local exploitation and exploration).
Max Iterations	T	500	Maximum number of iterations to run the optimization algorithm.
Crossover Probability	pc	0.7	Probability for genetic crossover operation in evolving sparrow positions.
Mutation Probability	pm	0.01	Probability for genetic mutation operation to maintain diversity in the population.

- Opposition-based learning is applied during population initialization to improve diversity, avoiding premature convergence.
- The elite opposition-based learning selects a subset of elite individuals for generating opposite solutions, ensuring exploration of promising regions.
- Sparrow behavior is modeled with producers, scroungers, and guards with proportions ND and SG regulating their counts.
- The integrated Genetic Algorithm component’s crossover and mutation probabilities are adjusted using golden ratio-inspired adjustment formulas for quicker convergence, balancing exploration and exploitation.
- These parameter settings were chosen empirically to balance computational cost and effectiveness, resulting in a variety of high-quality GUI test suites whose performance was verified by extensive experiments.
- When these parameters are combined, OOGSSA can effectively explore the GUI event space, produce a variety of test cases, and maximize coverage without sacrificing computational viability.

### Hardware and software setup

A strong computational infrastructure comprising an NVIDIA GeForce GTX 1080Ti GPU, an Intel Core i7 processor, and 32GB of RAM was used in the hardware and software configuration. The Python platform’s Terra Amazon was used to train and develop the model. Table 3 provides the hyperparameters for the Quasi Oppositional Genetic Sparrow Search Algorithm for effective GUI test case generation.

**Table 3:** Hyperparameters.

Hyperparameters	Values
Coverage goal	EFG
No. of iteration	500
Population size in each algorithm	30
Search agent	50
SG	0.3
ST	0.6
ND	0.4

OOGSSA’s hyperparameters were determined through empirical assessment and previous research on evolutionary optimization and swarm intelligence. To ensure sufficient diversity in the search space without imposing undue runtime costs, the population size (30) and search agent count (50) were selected in a way that balanced exploration and computational efficiency. After noticing that additional iterations produced only slight improvements in coverage metrics, the number of iterations (500) was set. The discovery rate (ND = 0.4) and safety threshold (ST = 0.6) were chosen in accordance with the Sparrow Search Algorithm’s general behavior, where moderate values promotes convergence stability while preventing premature stagnation. In similar, during search operations, the sparrows on guard ratio (SG = 0.3) guarantees an efficient trade-off between exploitation (scroungers) and exploration (producers). In order to achieve optimal diversity and consistent convergence in the generated GUI test suites, these parameter values were experimentally verified to support algorithmic robustness and performance reliability.

## Result and discussion

### Performance evaluation

The performance quality of the proposed Quasi Oppositional Genetic Sparrow Search Algorithm for the reliable GUI test case generation is assessed using the evaluation metrics listed below.

- Test suite size

It is used for the evaluation from a multi-objective perspective because it is a cost measure. This shows how many test cases the suggested GUI test case generation method produced overall. The test suite’s size is a crucial indicator of how thorough the testing process was. More extensive testing coverage is implied by a larger test suite, but the costs of test execution and maintenance are also higher.This objective function is represented as $\:{f}_{1}\left({x}^{\sim}\right)$ where $\:{x}^{\sim}$ is the solution. To increase the test suite size above 75, consider factors such as testing requirements, resource constraints, effectiveness of test cases, and prioritization of testing efforts. Table 4 represents the value of test suite size obtained.

**Table 4:** Evaluation of test suite Size.

Metric	Value
Size of the test suite	75

- Diversity of Test Suites

This measures the effectiveness of the testing community. Here we use two objective measures for the diversity of test suite.

- Jaccard similarity index

The similarity between pairs of test cases in the test suite is measured by Jaccard Similarity Index. A higher Jaccard Similarity Index indicates greater similarity among test cases. The mathematical expression of Jaccard Similarity is given below,

11 $$\:Jaccard\:Similarity,J(A,B)=\:\left|\frac{A\cap\:B}{A\cup\:B}\right|$$

where $\:\left|\frac{A\cap\:B}{A\cup\:B}\right|$ represents ratio of cardinality of the intersection of set A and B to the cardinality of the union of set A and B.

b. Dice–Jaro–Winkler dissimilarity

The dissimilarity between pairs of test cases in the test suite is measured by Dice–Jaro–Winkler Dissimilarity. A lower Dice–Jaro–Winkler Dissimilarity indicates greater similarity between test cases. Conversely, a higher value suggests greater diversity among the test cases. The mathematical expression is as follows.

12 $$\:{d}_{djw}=1-\left(\frac{({T}_{1}\cap\:{T}_{2})+df(1-{d}_{jw})}{({T}_{1}\cap\:{T}_{2})+w\left(\frac{n-df}{n}\right)}\right)$$

A Jaccard Similarity Index close to 1 suggests that the test cases cover similar aspects of the GUI application, while an index closer to 0 indicates greater diversity among the test cases.

c. Cosine similarity

Cosine similarity measures the cosine of the angle between two vectors representing the test cases. It quantifies similarity in terms of direction regardless of magnitude, making it effective for comparing feature-based representations where frequency or intensity vary. It is given in Eq. (13). For Vector representation x and y:

13 $$\:Cosine\left( {x,y} \right) = \frac{{x.y}}{{\left\| x \right\|\left\| y \right\|}}$$

d. Sørensen–Dice coefficient

A similarity metric that doubles the weight of shared elements relative to the total size of both sets. It emphasizes commonality and is helpful when partial overlap matters more than overall set size differences, commonly used in information retrieval and ecology.

14 $$\:DSC\left(A,B\right)=\frac{2|A\cap\:B|}{\left|A\right|+\left|B\right|}$$

Similar to Jaccard but gives more weight to the shared elements.

e. Levenshtrin distance

Measures the minimum number of single-character edits (insertions, deletions, substitutions) needed to transform one sequence into another, normalized between 0 and 1. It captures sequence dissimilarity explicitly, valuable for test cases represented as ordered event sequences.

**Fig. 8:**
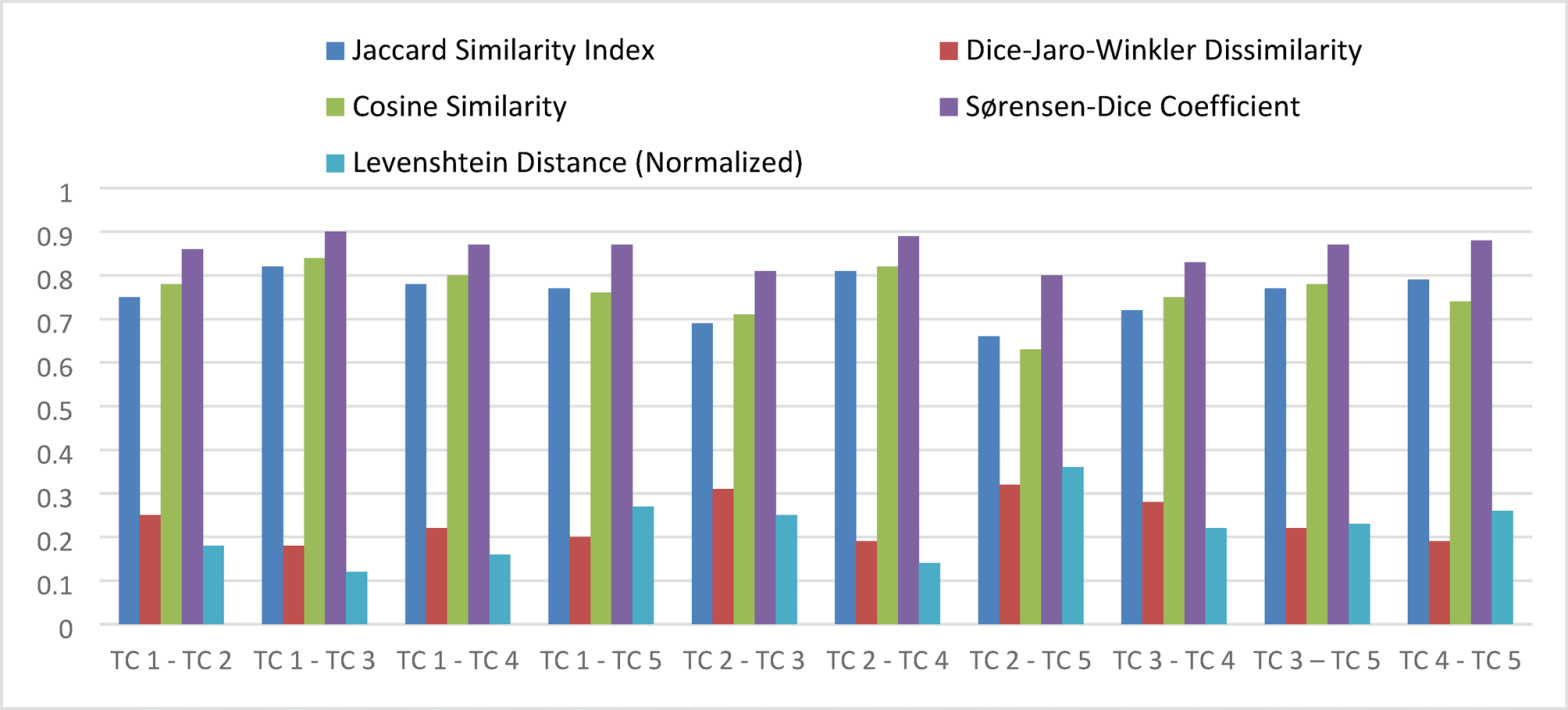
Graphical Representation of Similarity and Dissimilarity Index.

Table 5 represents the similarity and dissimilarity index obtained and Fig. 8 represents the graphical illustration.

**Table 5:** Evaluation of similarity and dissimilarity index.

Test case pair	Jaccard Similarity Index	Dice–Jaro–Winkler Dissimilarity	Cosine Similarity	Sørensen-Dice Coefficient	Levenshtein Distance (Normalized)
TC 1– TC 2	0.75	0.25	0.78	0.86	0.18
TC 1–TC 3	0.82	0.18	0.84	0.90	0.12
TC 1–TC 4	0.78	0.22	0.80	0.87	0.16
TC 1–TC 5	0.77	0.20	0.76	0.87	0.27
TC 2–TC 3	0.69	0.31	0.71	0.81	0.25
TC 2–TC 4	0.81	0.19	0.82	0.89	0.14
TC 2–TC 5	0.66	0.32	0.63	0.80	0.36
TC 3–TC 4	0.72	0.28	0.75	0.83	0.22
TC 3–TC 5	0.77	0.22	0.78	0.87	0.23
TC 4–TC 5	0.79	0.19	0.74	0.88	0.26

- Mouse event coverage

Mouse event coverage refers to the extent to which mouse events, such as clicks, drags, and hovers, are exercised or covered by the test cases in a test suite compared to all possible mouse events in the GUI application. It is an important metric in GUI testing as it measures how well the test suite evaluates the interactive functionalities of the software application. The mathematical expression is as follows:

13 $$\:Mouse\:Event\:Coverage\:\left(\%\right)=\frac{No:\:of\:covered\:Mouse\:Events}{Total\:No.\:of\:Possible\:Mouse\:Events}$$

- Widget coverage

Measures the proportion of GUI widgets (buttons, menus, text fields, sliders, etc.) exercised by the test cases. The mathematical expression was given in Eq. (14). This metric helps ensure that all components of the interface are tested.

14 $$\:Widget\:Coverage=\frac{Number\:of\:accessed\:widgets}{Total\:EquationNumber\:of\:widgets\:in\:GUI}\times\:100$$

- Event-flow coverage

Evaluates how many event transitions (from one GUI state to another) are covered in the test suite.Based on the Event Flow Graph (EFG) was already constructed in the study. The mathematical expression was given in Eq. (15).

15 $$\:Event-Flow\:Coverage=\frac{Covered\:event\:transitions}{Total\:Transitions\:in\:EFG}\times\:100$$

- Window or screen coverage

Measures the portion of application windows or screens visited during testing.Particularly it is relevant for multi-window or modular GUI systems.

- Path coverage

It ensures that logical interaction sequences are validated by confirming the number of different GUI navigation paths or workflows that are tested.

- Keyboard event coverage

It is crucial for testing accessibility. It ensures usability for non-mouse users with shortcuts, text inputs, and navigation keys (Tab, Enter, etc.).

- Fault detection rate (FDR)

It calculates the number of GUI errors (crashes, layout errors, and misbehaviors) in relation to the total number of potential injected or expected errors. It offers a useful addition to coverage metrics.

- Bug detection rate (BDR)

This metric measure the portion of faults found by automated test cases in relation to the total number of faults known or injected in benchmarks. Equation (16) provided the mathematical expression.

16 $$\:BDR=\frac{Number\:of\:bugs\:detected\:by\:the\:test\:suite}{Total\:known\:bugs\:in\:the\:application}\times\:100$$

The event coverage value obtained is shown in Table 6.

**Table 6:** Evaluation of similarity and dissimilarity index.

Metric	Value (%)
Mouse Event Coverage	95
Widget Coverage	88
Event-Flow Coverage	82
Window/Screen Coverage	91
Path Coverage	76
Keyboard Event Coverage	72
Bug Detection Rate (BDR)	85

The multi-dimensional coverage metrics show how thoroughly OOGSSA exercises various GUI features. The model performs well across complementary metrics, and mouse event coverage reaches 95%, indicating thorough interactive behavior testing. While event-flow coverage of 82% verifies navigation through the Event Flow Graph, widget coverage of 88% indicates that the majority of GUI components are accessed. While keyboard event coverage of 72% indicates potential for improvement in accessibility-focused testing, path coverage of 76% indicates the algorithm’s capacity to investigate intricate user workflows. Collectively, these metrics offer a comprehensive evaluation of the test suite’s efficacy that goes beyond single-event interaction, addressing issues with the thoroughness of the evaluation criteria. The bug detection rate (BDR) is used in addition to coverage metrics to assess the efficacy of the suggested OOGSSA method. The generated test suite achieved a BDR of 85% using a benchmark GUI with injected faults, demonstrating high capability in identifying faults beyond mere coverage. This evidence supports the usefulness of OOGSSA in enhancing software quality by both thoroughly examining GUI event spaces and successfully exposing hidden flaws.

### Statistical analysis

We carried out in-depth statistical analyses on important performance metrics, such as widget coverage, event-flow coverage, and fault detection rate, in order to fully assess the performance of the suggested OOGSSA framework. We obtained information about the consistency and variability of the results by repeating each experiment multiple times and calculating average values and standard deviations. For each metric, we first calculated descriptive statistics, specifically the means and standard deviations. Next, we used a one-way Analysis of Variance (ANOVA) test separately for each metric to determine whether the differences found across three distinct application frameworks the Online Login Portal built with Django, the E-Commerce Cart using React and Node.js, and a Survey Form Interface developed with HTML5 and JavaScript were significant. ANOVA examined the null hypothesis, which states that the average metric values for every application were the same. To determine precisely which pairs of apps differed significantly, we used Tukey’s Honest Significant Difference (HSD) test whenever we discovered significant differences (with a p-value less than 0.05). To ensure that our procedure is reliable and easily repeatable, these analyses were performed using common statistical software such as MATLAB’s Statistics Toolbox or Python’s SciPy and statsmodels libraries. We were able to confidently determine the reliability of the coverage improvements and fault detection rates by setting the significance level at 0.05 for all tests (Table 7 .

**Table 7:** Statistical analysis study across web GUIs.

Application framework	Widget coverage (%)	Event-flow coverage (%)	Fault detection rate (%)
Online Login Portal (Django)	86.1 ± 1.5	80.2 ± 1.8	91.0 ± 1.2
E-Commerce Cart (React + Node.js)	89.3 ± 1.2	83.0 ± 1.6	93.2 ± 1.1
Survey Form Interface (HTML5 + JS)	87.0 ± 1.7	78.4 ± 2.0	90.1 ± 1.5

For widget coverage, event-flow coverage, and fault detection rate metrics, independent ANOVA tests were performed for each of the three application frameworks. For instance, the ANOVA result for widget coverage showed an F-statistic of 8.42 with a p-value of 0.004, suggesting that the frameworks’ mean widget coverage differed statistically significantly at the 0.05 significance level. The E-Commerce Cart framework’s widget coverage was significantly higher than the Survey Form Interface’s (*p* = 0.03), according to subsequent Tukey’s HSD post-hoc tests. Metrics related to fault detection rate and event-flow coverage also showed notable variations. These findings support the robustness and flexibility of the method by confirming that OOGSSA’s performance significantly varies depending on the GUI platform.

### Computational complexity analysis

The suggested Opposition-based Opposition-Guided Sparrow Search Algorithm (OOGSSA) is evaluated for computational efficiency in terms of both time and space complexity. The algorithm is essentially made up of several modular stages that each contribute differently to the total cost.

- Time complexity

The time complexity of OOGSSA depends predominantly on the number of GUI elements, event transitions, population size, search dimensionality, and iteration count. The major stages contributing to computational effort are summarized in Table 8.

**Table 8:** Time Complexity.

Stage	Description	Approximate complexity
GUI Ripper and EFG Construction	Extraction of GUI widgets and event transitions. Forn GUI elements and m events, constructing the Event Flow Graph (EFG) requires traversal and edge linkage operations.	*O(n + m)*
Population Initialization (with EOBL)	Random initialization of N sparrow positions (test cases), each of dimensionality D, followed by generation of their opposite solutions.	*O(N×D)*
Fitness Evaluation	Each sparrow (test case) is evaluated based on multiple objectives, including coverage, diversity, and test sequence size. If each fitness computation takes O(D)O(D)O(D), the total becomes O(N×D)O(N \times D)O(N×D).	*O(N×D)*
Sparrow Search Update (SSA)	At every iteration, producer, scrounger, and aware sparrow positions are updated. With Titerations, the update complexity scales linearly as O(T×N×D)O(T \times N \times D)O(T×N×D).	*O(T×N×D)*
Genetic Operations (Crossover & Mutation)	Genetic operators are applied on a subset of individuals during each iteration. Crossover and mutation cost is linear in chromosome length, giving an additional cost of O(T×N×D)O(T \times N \times D)O(T×N×D).	*O(T×N×D)*
Elite Opposition-Based Learning (EOBL)	Reverse solutions are generated for the top k elite individuals in each iteration, incurring O(k×D)O(k \times D)O(k×D)time where k≪N.	*O(k×D)*

Thus, the dominant computational cost arises from the iterative optimization involving SSA and GA operations. The overall time complexity is therefore given by:.

$$\:O(T\times\:N\times\:D)$$

Where T = number of iterations, N = population size, and D = dimensionality (number of events per test case sequence). This complexity is comparable to that of standard swarm intelligence methods such as PSO, GA, and SSA, albeit with a slightly higher constant factor due to opposition-based learning and hybrid genetic operations. Nevertheless, the proposed OOGSSA attains faster convergence and improved optimization quality, reducing the effective number of iterations required to reach sub-optimal solutions in practice.

- Space complexity

The space complexity of OOGSSA arises mainly from storing the population, fitness values, and auxiliary parameters. The memory requirements of each component are given in Table 9.

**Table 9:** Space Complexity.

Component	Description	Complexity
Population Matrix	StoresNsparrow positions, each of dimensionD.	$\:O(N\times\:D)$
Fitness Vector	Contains fitness values associated with each sparrow.	$\:O\left(N\right)$
Opposition Population (EOBL)	Maintains elite and opposite individuals for diversity enhancement.	$\:O(k\times\:D)$
Auxiliary Variables	Includes temporary buffers for best/worst solutions, iteration counters, and search statistics.	$\:O(N+D)$

Consequently, the overall space complexity is $\:O(N\times\:D)$ indicating linear growth with respect to population size and test case dimensionality.

Compared with canonical SSA and GA, OOGSSA introduces additional operations due to quasi-oppositional learning and hybrid crossover–mutation schemes. Although this increases per-iteration computational cost marginally, the algorithm compensates through substantially faster convergence to global optima. In contrast, exhaustive GUI test generation whose complexity grows exponentially with event combinations $\:O\left({E}^{n}\right)$ is reduced to a polynomial-linear form by the search-based heuristic of OOGSSA, making it efficient and scalable for large GUI systems.The proposed OOGSSA exhibits high computational efficiency and scalability for GUI test case generation. Its time complexity $\:O(T\times\:N\times\:D)$ and space complexity $\:O(N\times\:D)$ reflect linear growth with controllable parameters, offering a favorable trade-off between computational cost and convergence reliability. The hybrid and opposition-based learning mechanisms ensure rapid and stable convergence toward high-quality test suites across complex event-driven systems.

### Discussion

In automated GUI testing across diverse software platforms and programming languages, the OOGSSA method demonstrates significant robustness and versatility. Its ability to function at the GUI event level, utilizing interactions like mouse clicks, keystrokes, and GUI component events without depending on language-specific source code analysis, is what mainly makes this possible. If programmatic access or modeling of GUI elements is available, OOGSSA enables smooth adaptation across desktop, web, and mobile environments by abstracting user interactions through platform-independent GUI models like event-flow graphs. Because its parameters, like population size, iteration count, and mutation probabilities, are modular and tunable, it can be tailored to a variety of platform complexities, improving scalability.

According to empirical findings, OOGSSA attains high coverage metrics, such as keyboard event coverage of 72%, mouse event coverage of 95%, widget coverage of 88%, and event-flow coverage of 82%. Crucially, an 85% success rate in identifying injected errors in controlled GUI environments is demonstrated by the bug detection rate metric. Together, these results demonstrate the method’s ability to produce varied and condensed test suites that not only cover a broad range of GUI interaction scenarios but also identify actual errors, highlighting its usefulness in enhancing software quality.

However, the current evaluation’s immediate generalizability to larger, real-world software systems is limited because it is primarily based on a controlled calculator-based GUI. It is still a significant challenge to expand evaluation to a range of open-source and commercial web applications with intricate multi-window, asynchronous, and dynamic UI behaviors. Convergence speed and fault detection may be impacted by non-trivial state explosion, intricate event dependencies, and hitherto undiscovered interaction patterns introduced by such heterogeneous environments. Furthermore, while mouse events serve as the basis for interaction coverage, achieving truly holistic testing coverage requires a greater focus on other modalities, such as thorough keyboard input and touch gestures that are crucial for accessibility and mobile platforms. Further optimization is required due to the inherent computational cost of metaheuristic algorithms such as OOGSSA, particularly when scaling to large GUI state spaces or long interaction sequences. Adaptive or self-tuning techniques could increase performance stability and efficiency, but parameter tuning is still empirical. Test relevance and completeness would also be improved by incorporating platform-specific metrics like cross-browser consistency, responsiveness, and accessibility compliance.

#### Future work

In order to thoroughly validate scalability, robustness, and fault detection capability in real-world scenarios, future research aims to expand OOGSSA’s evaluation to a curated set of real-world web and mobile applications encompassing diverse frameworks such as React, Angular, and native mobile platforms. In order to improve coverage criteria, especially for cross-platform and assistive technology-inclusive testing, efforts will also concentrate on adding additional interaction types, such as keyboard events, touch gestures, and voice commands. Search space efficiency and computational overhead reduction will be the algorithmic goals of investigating hybrid metaheuristic combinations, dynamic parameter adaptation, and parallelization techniques. Improving fitness functions’ adaptability to incorporate platform-specific quality attributes will enable more sophisticated optimization in line with changing user experience standards.Lastly, seamless adoption in continuous integration pipelines will be made possible by integrating OOGSSA with well-known automation frameworks like Selenium, Appium, and Robot Framework. This will lead to more automated, precise, and effective GUI test generation practices across the industry.

## Conclusion

Graphical user interface (GUI) testing has advanced significantly with the introduction of the Quasi Oppositional Genetic Sparrow Search Algorithm (OOGSSA). To guarantee software dependability and user satisfaction, there is an urgent need for effective and thorough testing methodologies due to the growing complexity of GUI-based applications. In order to provide dependable test cases for GUI applications, the approach combines components of SBST and MBST, genetic algorithms (GA), and Sparrow Search Algorithm (SSA). OOGSSA optimizes the search process by utilizing opposition-based learning techniques and sparrow foraging behavior, which results in the creation of varied and efficient test suites.

By promoting the selection of individuals based on fitness values and enabling crossover and mutation operations to more efficiently explore the solution space, the algorithm’s integration of GA further improves population quality. The performance evaluation’s findings show how effective OOGSSA is at creating GUI test cases. High diversity metrics, such as the Jaccard Similarity Index and Dice–Jaro–Winkler Dissimilarity, show thorough coverage of GUI functionalities while preserving test case diversity with a test suite size of 75. Furthermore, attaining a mouse event coverage of 95% highlights how well our method works for assessing interactive features that are essential to user experience. Essentially, the OOGSSA model provides a viable way to improve the quality and dependability of GUI-based software applications while also addressing the difficulties associated with GUI testing. Techniques like OOGSSA will be essential to guaranteeing the smooth operation and usability of contemporary graphical user interfaces across a range of platforms and devices as technology develops.

## Data Availability

The datasets used and/or analysed during the current study available from the corresponding author on reasonable request.

## References

[1] Jansen, B. J. The graphical user interface. *ACM SIGCHI Bull.* **30** (2), 22–26 (1998).

[2] Aberg, G. & Chang, J. Applying cognitive science research in graphical user interface (GUI). *Umea Inst. Des.* 23–28 (2005).

[3] Oulasvirta, A., Dayama, N. R., Shiripour, M., John, M. & Karrenbauer, A. Combinatorial optimization of graphical user interface designs. *Proc. IEEE*. **108** (3), 434–464 (2020).

[4] Su, T., Wang, J. & Su, Z. Benchmarking automated gui testing for android against real-world bugs. In *Proceedings of the 29th ACM Joint Meeting on European Software Engineering Conference and Symposium on the Foundations of Software Engineering*, 119–130. (2021).

[5] Yoon, J., Feldt, R. & Yoo, S. Intent-driven mobile GUI testing with autonomous large language model agents.

[6] Bons, A., Marín, B., Aho, P. & Vos, T. E. Scripted and scriptless GUI testing for web applications: an industrial case. *Inf. Softw. Technol*. **158**, 107172 (2023).

[7] Ricós, F. P., Slomp, A., Marín, B., Aho, P. & Vos, T. E. Distributed state model inference for scriptless GUI testing. *J. Syst. Softw.* **200**, 111645 (2023).

[8] Nie, L., Said, K. S., Ma, L., Zheng, Y. & Zhao, Y. A systematic mapping study for graphical user interface testing on mobile apps. *IET Softw.* **17** (3), 249–267 (2023).

[9] Marculescu, B., Feldt, R., Torkar, R. & Poulding, S. Transferring interactive search-based software testing to industry. *J. Syst. Softw.* **142**, 156–170 (2018).

[10] Zayed, H. A. B. & Maashi, M. S. Optimizing the software testing problem using Search-Based software engineering techniques. *Intell. Autom. Soft Comput.* **29**(1) (2021).

[11] Balera, J. M. & de Santiago Júnior, V. A. A systematic mapping addressing hyper-heuristics within search-based software testing. *Inf. Softw. Technol.* **114**, 176–189 (2019).

[12] Yan, J. et al. Efficient testing of GUI applications by event sequence reduction. *Sci. Comput. Program.* **201**, 102522 (2021).

[13] Coppola, R., Morisio, M., Torchiano, M. & Ardito, L. Scripted GUI testing of android open-source apps: evolution of test code and fragility causes. *Empir. Softw. Eng.* **24**, 3205–3248 (2019).

[14] Lugaresi, G. & Matta, A. Automated digital twin generation of manufacturing systems with complex material flows: graph model completion. *Comput. Ind.* **151**, 103977 (2023).

[15] Balaha, H. M. & Hassan, A. E. S. Skin cancer diagnosis based on deep transfer learning and sparrow search algorithm. *Neural Comput. Appl.* **35** (1), 815–853 (2023).

[16] Reddy, C. K. K. et al. Twined ensemble framework for network security: integrating random Forest, AdaBoost, and gradient boosting for enhanced intrusion detection. *Discover Internet Things*. **5** (1), 107 (2025).

[17] Anand, R. P., Senthilkumar, V., Kumar, G., Rajendran, A. & Rajaram, A. Dynamic link utilization empowered by reinforcement learning for adaptive storage allocation in MANET. *Soft Comput.-A Fusion Found. Methodol. Appl.* **28**(6) (2024).

[18] Kannan, S. & Rajaram, A. QoS aware power efficient multicast routing protocol (QoS-PEMRP) with varying mobility speed for mobile ad hoc networks. *Int. J. Comput. Appl. ***60**(18) (2012).

[19] Maheswari, J. et al. I/O Match (I/O Mat) and behavioral match (Beh Mat) based semantic web service discovery. *J. Theor. Appl. Inf. Technol. ***69**(2) (2014).

[20] Rajaram, A. & Joseph, S. Cluster based neighbor coverage routing scheme for manet. *J. Theor. Appl. Inf. Technol. ***68**(3) (2014).

[21] Samal, U. & Kumar, A. Incorporating human dynamics into software reliability analysis: learning, fatigue, and efficiency considerations. *Int. J. Syst. Assur. Eng. Manag.* 1–10 (2024).

[22] Samal, U. & Kumar, A. Fault removal efficiency: A key driver in software reliability growth modeling. In *Reliability Engineering for Industrial Processes: an Analytics Perspective*, 95–106 (Springer Nature Switzerland, 2024).

[23] Samal, U. & Kumar, A. An enhanced software reliability growth model considering dynamic fault removal efficiency and residual error change rate. *J. Inf. Sci. Eng.* **40**(6) (2024).

[24] Aghdam, Z. K. & Arasteh, B. An efficient method to generate test data for software structural testing using artificial bee colony optimization algorithm. *Int. J. Software Eng. Knowl. Eng.* **27** (06), 951–966 (2017).

[25] Shomali, N. & Arasteh, B. Mutation reduction in software mutation testing using firefly optimization algorithm. *Data Technol. Appl.* **54** (4), 461–480 (2020).

[26] Arasteh, B., Hosseini, S. M. J. & Traxtor An automatic software test suit generation method inspired by imperialist competitive optimization algorithms. *J. Electron. Test.* **38**, 205–215. 10.1007/s10836-022-05999-9 (2022).

